# Galectin-3 promotes secretion of proteases that decrease epithelium integrity in human colon cancer cells

**DOI:** 10.1038/s41419-023-05789-x

**Published:** 2023-04-13

**Authors:** Shun Li, David Mark Pritchard, Lu-Gang Yu

**Affiliations:** 1 Department of Biochemistry and Systems Biology, Institute of Systems, Molecular and Integrative Biology, University of Liverpool, Liverpool, UK; 2 Department of Molecular and Clinical Cancer Medicine, Institute of Systems, Molecular and Integrative Biology, University of Liverpool, Liverpool, UK

**Keywords:** Cancer microenvironment, Glycobiology

## Abstract

Galectin-3 is a galactoside-binding protein that is commonly overexpressed in many epithelial cancers. It is increasingly recognized as a multi-functional, multi-mode promoter in cancer development, progression, and metastasis. This study reports that galectin-3 secretion by human colon cancer cells induces cancer cell secretion, in an autocrine/paracrine manner, of a number of proteases including cathepsin-B, MMP-1 and MMP-13. The secretion of these proteases causes disruption of epithelial monolayer integrity, increases its permeability and promotes tumour cell invasion. This effect of galectin-3 is shown to be mediated through induction of cellular PYK2-GSK3α/β signalling and can be prevented by the presence of galectin-3 binding inhibitors. This study thus reveals an important mechanism in galectin-3-mediated promotion of cancer progression and metastasis. It provides further evidence to the increased realization of galectin-3 as a potential therapeutic target for the treatment of cancer.

## Introduction

Proteases are a family of proteolytic enzymes that play fundamental roles in the progression and metastasis of cancers of epithelial origins [1]. The concentrations of proteases are generally low in physiological conditions, but are substantially increased in cancer [2]. Higher amounts of proteases in the tumour microenvironment promote tumour cell invasion at the primary tumour site and increase tumour cell metastasis to distant organs [2].

Proteases are broadly classified into seven groups according to their catalytic actions: aspartic, glutamic, metalloproteases, serine, cysteine, threonine, and asparagine peptide lyases. Proteases in the first three groups use a water molecule as a nucleophile to attack the peptide bond of the substrate, while the other groups of proteases use an amino acid residue as a nucleophile. Each group of proteases acts on different protein substrates and is involved in regulating different stages in the progression of cancer. For example, cysteine proteases (e.g. the cathepsin family of proteases) can degrade both intracellular and extracellular matrix (ECM) proteins [3], and assist tumour cell invasion into nearby tissues, blood and lymph vessels [4]. Serine proteases can degrade extracellular matrix proteins as well as growth factors such as epidermal growth factor (EGF), fibroblast growth factor-2 (FGF-2) and hepatocyte growth factor/scatter factor (HGF-SF) [5] and are involved in tumour cell invasion, angiogenesis and metastasis [6]. Aspartic proteases, which have two highly conserved aspartates in their catalytic site [7], can cleave chemokines and reduce the anti-tumoural immune response and promote cancer cell migration [8]. They can also cleave natural protease inhibitory proteins, such as plasminogen activator inhibitor-1, and stimulate the activation of plasminogen activators in favour of cancer progression [9]. Threonine proteases are normally the catalytic subunits of the proteasome which degrades a variety of proteins through polyubiquitination [10]. Matrix metalloproteases (MMPs) primarily catalyse the degradation of ECM, but can also act on a range of other substrates such as growth factors and other proteases [11]. Many MMP members such as MMP-1, MMP-2, and MMP-9 are overexpressed in cancer [12] and contribute to cancer cell growth, apoptosis, angiogenesis, invasion and metastasis [13].

Galectin-3 is a β-galactoside-binding protein and is overexpressed in various types of epithelial cancer including colorectal, breast, lung, prostate, pancreatic, head and neck cancer and melanoma [14]. Galectin-3 overexpression is often associated with metastasis and poor prognosis [15]. Higher levels of circulating galectin-3 are seen in cancer patients, in particularly those with metastases [16]. Many studies have shown that overexpression of galectin-3 promotes multiple steps in cancer progression and metastasis such as cancer cell adhesion, invasion, angiogenesis and immune suppression [17]. Many of these cancer-promoting actions of galectin-3 have shown to be associated with galectin-3 interaction with galactoside-terminated cell surface glycoproteins. For example, binding of galectin-3 to the oncofoetal Thomsen-Friedenreich Galβ1,3GalNAcα-Thr/Ser (TF antigen) on the transmembrane mucin protein MUC1 [18] enhances circulating tumour cell homotypic aggregation and survival [19] and increases tumour cell heterotypic adhesion to vascular endothelium [20]. Binding of galectin-3 to CD146 via N-linked glycans on the endothelial cell surface enhances secretion of the metastasis-promoting cytokines IL-6 and G-CSF [18]. The broad influence of galectin-3 on promotion of cancer progression led us to hypothesise that galectin-3 overexpression and secretion by cancer cells may influence the cellular secretion of proteases and thus contribute to tumour cell invasion and metastasis. A series of studies was conducted in this study to test this hypothesis and the results strongly support this notion.

## Materials and methods

### Materials

Crystal violet solution (1%) and SIGMAFAST™ OPD were obtained from Sigma (Gillingham, UK). Bovine Serum Albumin (BSA) was obtained from Tocris (Bristol, UK). Matrixgel matrix® (phenol red-free), Trans-wells with 8.0 µm PET membrane and 0.4 µm polyester membrane were purchased from Corning (ME, USA). Biotinylated-anti-galectin-3 (BAF1154) antibody and antibodies against galectin-3 (MAB1154), STAT1 (MAB1490), Phospho-STAT1(MAB2894), STAT3 (MAB1799), Phospho-STAT3 (MAB4934), PYK2 (AF4589), phosphor-PYK2 (MAB6210), GSK3α/β (AF2157), Phospho-GSK3α/β (AF1590); Proteome Profiler Human Protease Array (ARY021B), Proteome Profiler Human Phospho-Kinase Array (ARY003C); Human MMP-13 DuoSet ELISA (DY511), Human Cathepsin-B DuoSet ELISA (DY2176) PYK2 inhibitor PF-431396 (4278) and GSK3α/β inhibitor SB 216763 (1616/1) were all purchased from R&D Systems (Abingdon, UK). Peroxidase-conjugated secondary antibodies were purchased from Cell signalling (Massachusetts, USA).

### Cells

Human colon cancer SW620, HCT116, Caco-2 and HT29 cells, were all obtained from European Collection of Cell Cultures (Salisbury, UK). All the cells (except HCT116) were cultured in Dulbecco′s Modified Eagle′s Medium (DMEM) (Gibco, Loughborough, UK) containing 200 mM l-glutamine, 0.4% penicillin and streptomycin and 10% foetal calf serum (FCS). HCT116 cells were cultured in McCoy’s medium (Gibco, Loughborough, UK) containing 200 mM l-glutamine, 0.4% penicillin and streptomycin and 10% foetal calf serum. The cell lines were last authenticated in 2020 by DNA profiling at the Cell Line Authentication Facility, University of Liverpool.

Galectin-3 knockdown SW620-shGal3 and control SW620-shCon cells were generated using galectin-3 shRNA and control shRNA from SW620 as previously described [21].

### Cell invasion

Trans-well inserts (0.8 µm pore size) in 24-well plates were coated with 100 µl Matrigel matrix proteins (20 µg/ml) for 2 h at 37 °C. After a gentle wash with phosphate buffered saline (PBS), they were introduced with 150 µl cell suspension (1 × 10^5^ cells/ml) containing 10 µg/ml recombinant galectin-3 or 10 µg/ml BSA (control) in 1% FCS medium. Five hundred µl culture medium containing 10% FCS was added to the bottom wells of the plates. After 16 h incubation at 37 °C, the matrix and uninvaded cells inside the inserts were gently removed with cotton swabs. The inserts were washed once with PBS and fixed in 2% formaldehyde/PBS for 20 min. After one wash with PBS, the inserts were stained with 0.5% crystal violet solution for 5–10 min. The cells at the bottom side of the membrane were then counted in three to five randomly selected fields of view (FOVs) under a microscope (Olympus B51, Olympus, Tokyo, Japan) with a ×20 objective.

### Measurement of proteases secretion by slot blot

Cells (1.5 × 10^6^) were cultured in six-well plates until they were 80–90% confluent. The cells were washed with PBS and introduced with serum-free medium containing 1% BSA with different concentration of galectin-3 or BSA. After 24 h culture, the medium was collected, and 250 μl was loaded onto a slot blot. After one wash with PBS, the nitrocellulose membranes were incubated with blocking buffer (1% BSA/PBS) for 1 h at room temperature before incubation with antibodies against Cathepsin-B (20 μg/ml), MMP-13 (2 μg/ml), or MMP-1 (40 μg/ml) overnight at 4 °C. The blots were washed three times with 0.05% Tween-20 in PBS and incubated with peroxidase-conjugated secondary antibody (1:5000) for 1 h. After six washes with 0.05% Tween-20 in PBS, the blots were developed using chemiluminescence SuperSignal™ kit (Thermo Fisher, Warrington, UK) and visualized with Molecular Imager® Gel Doc™ XR System (Biorad, Hempstead, UK). The density of the blots was quantified using Imagelab version 3.0.1 (Biorad, Hempstead, UK).

### Measurement of protease secretion by ELISA

Cells (1 × 10^6^) were cultured in 6-well plates until 80–90% confluence. The cells were washed with PBS and introduced with serum-free medium containing 1% BSA without or with galectin-3 (10 μg/ml), lactose (100 μM) or asialofetuin (ASF) (20 μg/ml) for different times at 37 °C. The culture medium was collected, centrifuged at 5000 rpm for 5 min and the concentrations of proteases or galectin-3 in the supernatants were analysed by ELISA kits according to the manufacturer’s protocol.

### Immunoblotting

Cellular proteins were separated by SDS-PAGE followed by electro-transfer onto nitrocellulose membranes. The membranes were incubated with blocking buffer (1% BSA/PBS) for 1 h before incubation with antibodies against phospho-PYK2 (0.5 µg/mL), phospho-GSK3α/β (0.2 µg/mL), phospho-STAT1 (0.5 µg/mL), phospho-STAT3 (0.5 µg/mL), MMP-1 (1 µg/mL), MMP-13 (0.5 µg/mL), or Cathepsin-B (0.5 µg/mL) overnight at 4 °C. The blots were washed three times with 0.05% Tween-20 in Tris-buffered saline (TBS) before incubation with peroxidase-conjugated secondary antibody (1:5000) for 1 h. After six washes with 0.05% Tween-20 in TBS, the blots were developed using chemiluminescence SuperSignal™ kit and visualized with Molecular Imager® Gel Doc™ XR System. The blots were stripped by stripping buffer (Tris-HCl 62.5 mM, Mercaptoethanol 100 mM and SDS 2%) and reprobed with antibodies against PYK2 (1 µg/mL), GSK3α/β (0.1 µg/mL), STAT1 (1 µg/mL), or STAT3 (0.1 µg/mL). The density of the protein bands was quantified using Imagelab.

### Human protease profile array

Fifty-percent confluent cells were incubated with 10 µg/ml galectin-3 or BSA for 24 h at 37 °C in serum-free medium. The culture medium was collected and centrifuged at 5000 rpm for 10 min and the levels of 35 common proteases were analysed by Proteome Profiler Human Protease Array according to the manufacturer’s protocols. The density of the blots was quantified by Imagelab Software.

### Human protein kinase profile array

Cells were seeded at 1 × 10^6^ cells/ml into 6-well plate and cultured to 80% confluence. The cells were washed three times with PBS and introduced with serum-free medium containing 1% BSA with 10 μg/ml galectin-3 or 10 μg/ml BSA for 30 min at 37 °C. The cells were washed three times with 0.05% Tween-20/PBS and lysed in SDS-sample buffer. The phosphorylation Levels of 37 most common kinases in cell signalling were analysed by the Proteome Profiler Human Phospho-Kinase Array according to the manufacturer’s protocols.

### Assessments of cell–cell junction integrity and permeability

Caco-2 cells (5 × 10^5^/ml) were seeded into 0.4 μm pore size trans-wells and cultured for various times at 37 °C for cell monolayer formation. The monolayer integrity was measured with an ohmmeter until transepithelial electrical resistance (TEER) reached plateau (approximately 3000 Ωcm^2^). HCT116, SW620-shGal3/SW620-shCon cells (1 × 10^5^/ml) were cultured in 24-well plates for 3 days at 37 °C. The culture medium was collected and centrifuged at 5000 rpm for 5 min and used as conditioned media (CM). The culture medium in the trans-wells was removed and replaced with 0.5 ml/well CM. The cells in trans-wells were cultured at 37 °C for various times and monolayer integrity was measured with an ohmmeter.

For assessment of the cell monolayer permeability, 1 mg/ml FITC-dextran (20 kDa) was introduced to the tight cell monolayers (TEER, approximately 3000 Ωcm^2^) in the trans-wells for 30 min at room temperature. The culture medium in the bottom wells was collected and centrifuged at 5000 rpm for 5 min and the fluorescence intensity was measured using a fluorescence microplate reader GENios Plus (TECAN, Reading, UK).

### Statistical analysis

One-way analysis of variance (ANOVA) followed by Bonferroni correction were used for multiple comparisons. Differences were considered significant when *P* < 0.05.

## Results

### Galectin-3 induces protease secretions in human colon cancer cells

**Fig. 1:**
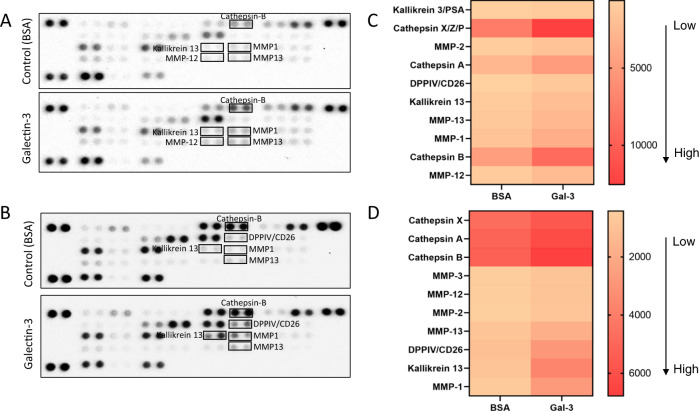
Galectin-3 induces the secretion of a number of proteases in human colon cancer cells. SW620 (**A**) and HCT116 (**B**) cells were treated with 10 µg/ml galectin-3 or BSA for 24 h before the levels of 35 proteases were analysed by Proteome Profiler Human Protease Array. The ten most changed proteases by the cells in response to galectin-3 are shown in heatmaps in (**C**) (SW620) and **D** (HCT116) (with changes ranked from lowest to highest).

To assess effect of galectin-3 on protease secretion from cancer cells, human colon cancer SW620 and HCT116 cells were first analysed with protease arrays. SW620 and HCT116 cells were cultured in the presence or absence of 10 μg/ml galectin-3, a concentration that is close to that seen in the circulation of colon cancer patients with metastasis [22] for 24 h, and the concentrations of proteases in the media was measured by the Proteome Profiler Human Protease Array which covers 35 common proteases. In comparison to cells treated with control BSA, treatment of the cells with galectin-3 resulted in increased secretion of several proteases from both SW620 and HCT116 cells (Fig. 1A, B). Among the 35 proteases, 10 proteases showed increases in the galectin-3-treated cells in both SW620 and HCT116 cells in comparison to the controls (Fig. 1C, D). The five proteases shown the highest increases in response to galectin-3 were MMP-12 (2.25-fold), Cathepsin-B (2.04-fold), MMP-1 (2.04-fold), MMP-13 (1.97-fold) and Kallikrein 13 (1.94-fold) in SW620 cells and MMP-1 (4.66-fold), Kallikrein 13 (4.1-fold), DPPIV/CD26 (2.62-fold), MMP-13 (1.94-fold) and MMP-2 (1.93-fold) in HCT116 cells.

**Fig. 2:**
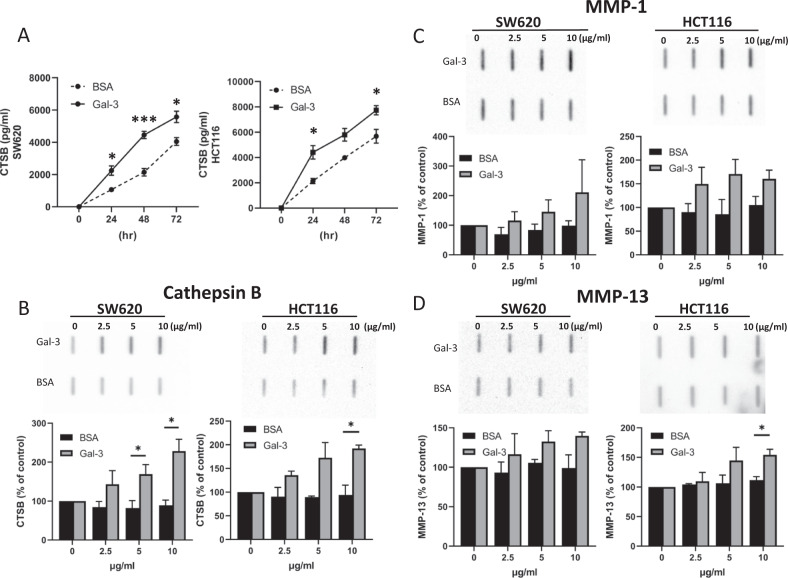
Galectin-3 induces protease secretion in human colon cancer cells in a dose- and time-dependent manner. SW620 and HCT116 cells were treated with galectin-3 or BSA (10 µg/ml) for different times before the concentrations of Cathepsin-B were analysed (**A**) by ELISA. SW620 and HCT116 cells were treated with different concentrations of galectin-3 for 24 h (**B**–**D**) before the levels of Cathepsin-B (**B**), MMP-1 (**C**), MMP-13 (**D**) in the medium were analysed by slot blotting. The slot densities from three independent experiments were quantified and are shown in the bottom panels. Data are presented as mean ± SD of three independent experiments, each in triplicate. ***P < 0.001, *P < 0.05 (ANOVA).

To further investigate the effect of galectin-3 on protease secretion, SW620 and HCT116 cells were treated with various pathological galectin-3 concentrations and the secretion of the three most highly affected proteases MMP-1, MMP-13 and cathepsin-B was analysed by ELISA and slot blotting. The presence of galectin-3 caused time- (Fig. 2A) and dose- (Fig. 2B) dependent increases in cathepsin-B secretion in both SW620 and HCT116 cells. After 24 h treatment with 10 μg/ml galectin-3, a 2.1- and 2.0-fold increase was seen in SW620 and HCT116 cells, respectively, when assessed by ELISA (Fig. 2A). When the secreted levels of these proteases in the culture medium were assessed by slot blotting, a 2.5- and 1.7-fold increase was seen in SW620 and HCT116 cells, respectively, after 24 h treatment with galectin-3 in comparison to control cells (Fig. 2B).

The levels of MMP-13 and MMP-1 secretion by SW620 and HCT116 cells were below the accurate measurement threshold by ELISA. Therefore, the levels of those proteases secreted to the culture media in cell response to different pathological galectin-3 concentrations were measured using slot blot. The presence of galectin-3 caused dose-dependent increases in MMP-13 and MMP-1 secretion in both SW620 and HCT116 cells (Fig. 2C, D). At 5 µg/ml, galectin-3 induced 1.25- and 1.36- fold increases of MMP-13 and 1.73- and 1.98- fold increases of MMP-1 secretion from SW620 and HCT116 cells, respectively. When cellular expression of these three proteases was analysed in SW620 and HCT116 cells by immunoblotting, their expression level in cells was not affected by the presence of galectin-3, except MMP-1 which showed a small 27% increase in cell response to treatment with 10 µg/ml (Supplemental Fig. S1). This indicates that galectin-3 predominantly enhances the secretion, but not the expression, of MMP-1, MMP-13, and cathepsin-B by those cells.

### Higher galectin-3 expression and secretion is associated with higher protease secretion in colon cancer cells

**Fig. 3:**
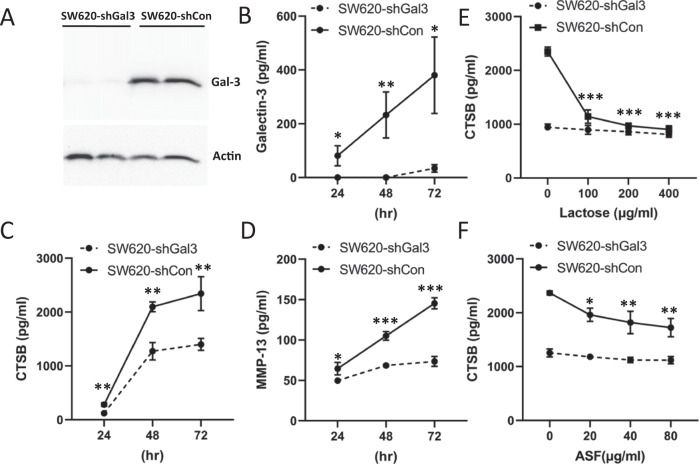
Galectin-3 expression and secretion by cancer cells promotes protease secretion. Amounts of galectin-3 expression and secretion in galectin-3 knockdown SW620-shGal3 and control SW620-shCon cells were assessed by immunoblotting (**A**) and galectin-3 ELISA (**B**). SW620-shCon cells secreted higher concentrations of cathepsin-B (**C**) and MMP-13 (**D**) than SW620-shGal3 cells when assessed by ELISA. The galectin-3-mediated cathepsin-B secretion was inhibited by the presence of galectin-3 inhibitors lactose (**E**) and ASF (**F**). Data are presented as mean ± SD from three independent experiments, each in triplicate. ***P < 0.001, **P < 0.01, *P < 0.05 (ANOVA).

To dissect the relationship between galectin-3 expression and protease secretion in the cells, galectin-3 expression in SW620 cells was suppressed using shRNA. Galectin-3 shRNA suppression led to 99% reduction of galectin-3 expression in the cells in comparison to transfection with control shRNA (Fig. 3A). Galectin-3 shRNA suppression was associated with significant reduction of galectin-3 secretion by the cells (Fig. 3B). Suppression of galectin-3 expression also resulted in significant reductions in Cathepsin-B and MMP-13 secretion (Fig. 3C, D). 40% (Fig. 3C) and 49% (Fig. 3D) lower levels of cathepsin-B and MMP-13 were produced by SW620-shGal3 than SW620-shCon cells after 72 h culture. The presence of galectin-3 inhibitors lactose (Fig. 3E) or asialofetuin (Fig. 3F) caused dose-dependent inhibition of cathepsin-B secretion from SW620-shCon cells but had no/less effect on SW620-shGal3 cells. This suggests that galectin-3-mediated reduction of protease secretion by the cells is predominately mediated by the extracellular action of galectin-3.

**Fig. 4:**
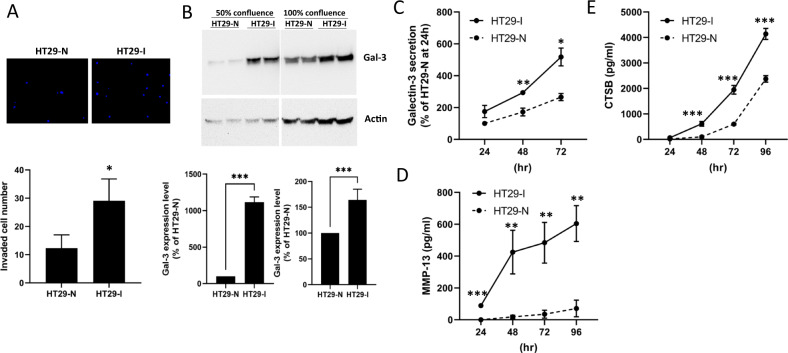
Galectin-3 expression and secretion promotes protease secretion and cell invasion of HT29 colon cancer cells. **A** HT29 cells were separated into invasive (HT29-I) and less/non-invasive (HT29-N) sub-populations. HT29-I cells express (**B**) and secrete (**C**) higher amounts of galectin-3 than the non-invasive HT29-N cells when assessed by immunoblotting (**B**) and ELISA (**C**). Representative blots from four experiments are shown in B (Top panels) with band densities shown as percentage galectin-3 expression (Bottom panels) HT29-I cells secrete higher amounts of MMP-13 (**D**) and CTSB (**E**) than HT29-N cells when assessed by ELISA. Data are presented as mean ± SD from three independent experiments, each in triplicate. ***P < 0.001, **P < 0.01, *P < 0.05 (ANOVA).

**Fig. 5:**
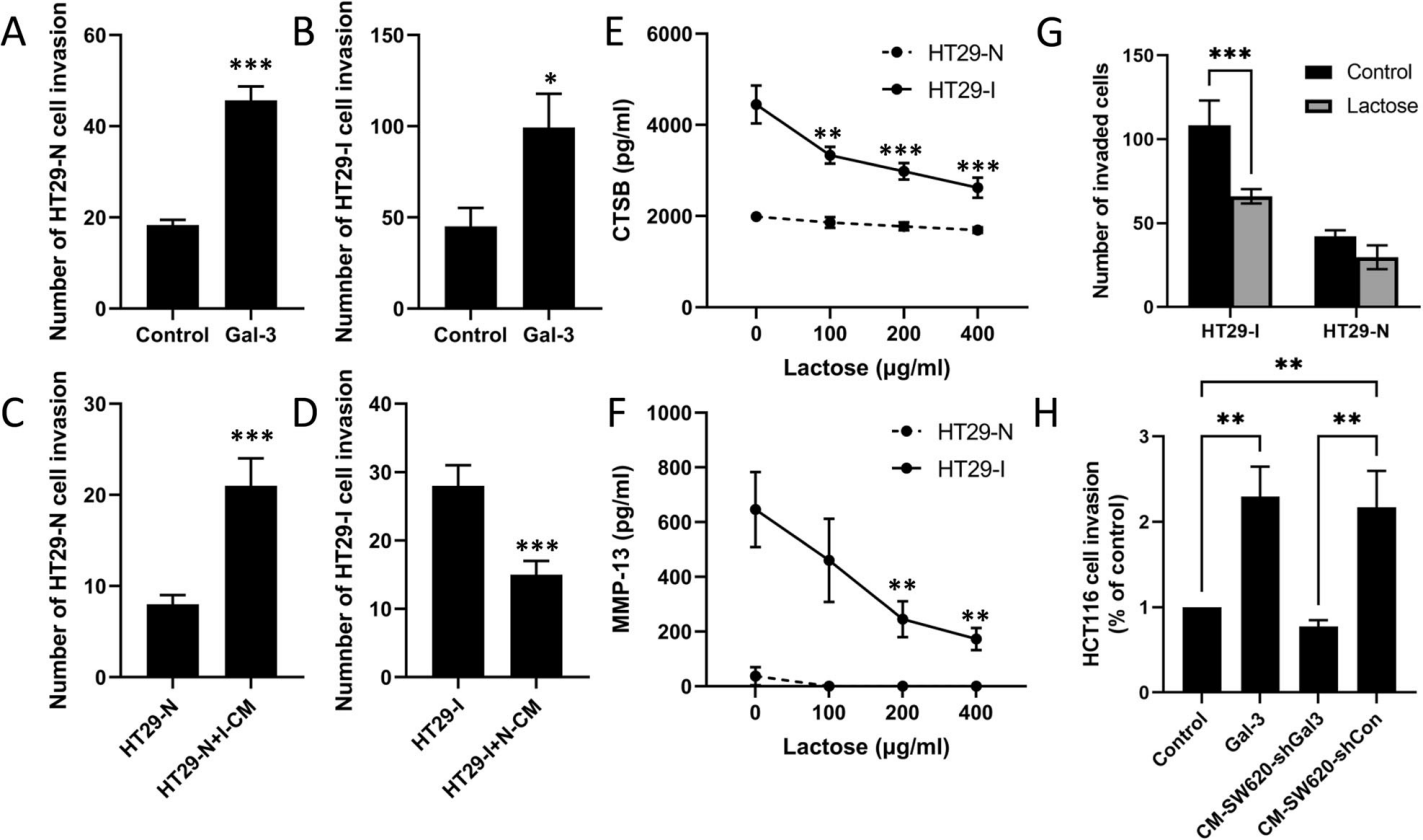
Galectin-3-mediated protease secretion increases cancer cell invasion. Introduction of exogenous galectin-3 increased invasion of both HT29-N (**A**) and HT29-I (**B**) cells. Replacing the culture medium in HT29-N cells with conditioned medium from HT29-I (I-CM) increased HT29-N cell invasion (**C**) while replacing the culture medium in HT29-I with conditioned medium from HT29-N (N-CM) led to reduction of HT29-I cell invasion (**D**). The presence of lactose deceased secretion of cathepsin-B (**E**) and MMP-13 (**F**) by HT29-I cells but had little effect on that of HT29-N cells. **G** The introduction of lactose inhibited the HT29-I cell invasion but had less effect on HT29-N cell invasion. **H** HCT116 cell invasion was assessed in the presence or absence of 10 µg/ml galectin-3, or with conditioned medium from SW620-shGal3 (CM-SW620-shGal3) and SW620-shCon (CM-SW620-shCon). Culture of the cells with galectin-3 or medium from CM-SW620-shCon, but not from CM-SW620-shGal3, increased HCT116 cell invasion. Data are presented as mean ± SD from three independent experiments, each in triplicate. ***P < 0.001, **P < 0.01, *P < 0.05 (ANOVA).

To further determine the relationship between galectin-3 expression and protease secretion, we separated the heterogenous HT29 colon cancer cells into invasive and none-invasive sub-populations by culturing the cells in trans-wells. The cells that remained in the trans-wells (non/less-invasive HT29-N) after 24 h culture and the cells which invaded to the bottom side of the trans-wells (invasive HT29-I) were collected. When these two sub-populations of HT29 cells were re-seeded into new trans-wells, HT29-I cells showed 2.4-fold higher invasion than HT29-N cells (Fig. 4A). The invasive HT29-I cells were found to express 12- and 1.55-fold, respectively, higher levels of galectin-3 than the non/less-invasive HT29-N cells in sub-confluent and fully confluent cells (Fig. 4B). HT29-I cells secreted significantly higher levels of galectin-3 than the HT29-N cells (Fig. 4C). At 72 h, a 1.9-fold higher level of galectin-3 was secreted by HT29-I than HT29-N cells. Much higher levels of MMP-13 (Fig. 4D) and Cathepsin-B (Fig. 4E) were secreted by HT29-I than HT29-N cells. At 72 h, 89% and 43% higher MMP-13 and Cathepsin-B levels were secreted by HT29-I cells than HT29-N cells. These results, together with the results demonstrated above with 1) the use of exogenous galectin-3 (Figs. 1 and 2), lactose and asialofetuin and (Fig. 3E, F) and conditioned medium of HT29-N and HT29-I (Fig. 5A–F), strongly suggest that galectin-3 expression and secretion by colon cancer cells promote cathepsin-B and MMP-13 secretion, autocrinely or paracrinely, and this effect of galectin-3 is predominately mediated through the extracellular action of galectin-3.

### Galectin-3-induced protease secretion increases cancer cell invasion

It is known that the presence of proteases in the tumour microenvironment can aid primary tumour cell invasion by digestion of basement proteins [23]. To determine the impact of galectin-3-mediated protease secretion on cancer progression, we assessed the influence of galectin-3-mediated protease secretion on tumour cell invasion through basement proteins. Introduction of 10 μg/ml exogenous galectin-3 to HT29 cells caused 2.5- and 2.2-fold, respectively, increases of invasion of HT29-N (Fig. 5A) and HT29-I (Fig. 5B) cells. When the culture medium of HT29-N cells was replaced with conditioned medium from HT29-I cells, which contains higher levels of proteases (Fig. 5C), HT29-N cell invasion was significantly increased (162%) in comparison to the cells without medium change (Fig. 5C). On the other hand, when the culture medium of HT29-I cells was replaced with conditioned medium from HT29-N cells, which contains less proteases (Fig. 5D), HT29-I cell invasion was significantly reduced (46%) in comparison to cells without medium change (Fig. 5D). Moreover, presence of the galectin-3 inhibitor lactose dose-dependently inhibited the secretion of cathepsin-B (Fig. 5E) and MMP-13 (Fig. 5F) as well as invasion (Fig. 5G) of HT29-I cells but had no significant effect on that of HT29-N cells.

Furthermore, when conditioned medium from SW620-shGal3 and SW620-shCon cells, which contain different amounts of galectin-3 (Fig. 3B), was used to assess the invasion of HCT116 cells, HCT116 cell invasion was significantly higher when cultured in the conditioned medium from SW620-shCon cells than in the conditioned medium from SW620-shGal3 cells (Fig. 5H). Again, introduction of exogenous galectin-3 significantly increased HCT116 cell invasion. Together, these results suggest that galectin-3-induced protease secretion promotes tumour cell invasion through basement proteins.

### Galectin-3-induced protease secretion disrupts cancer cell–cell contact and increases cell monolayer permeability and leakage

**Fig. 6:**
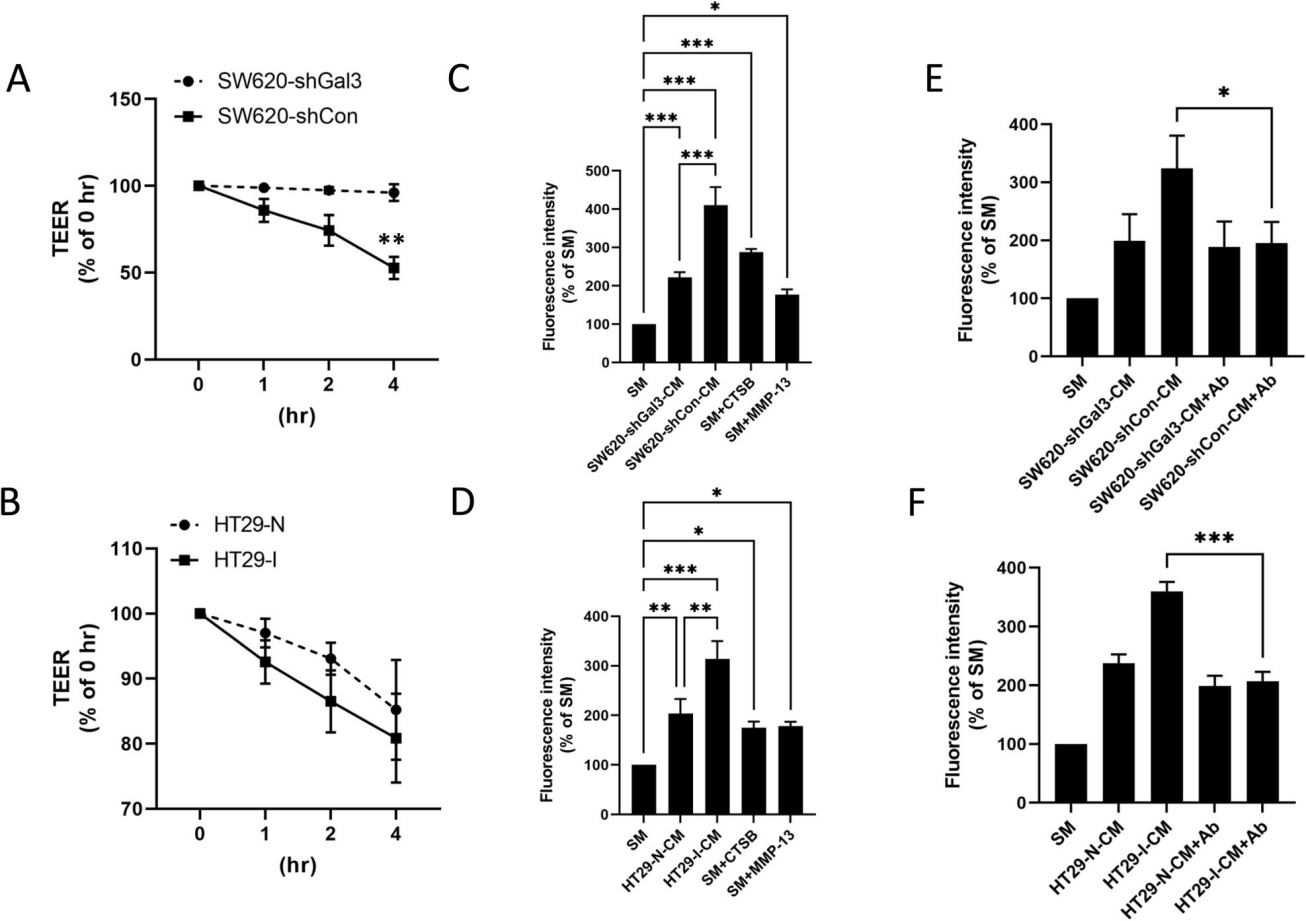
Galectin-3-induced protease secretion disrupts epithelial integrity and increases monolayer permeability of Caco-2 cells. Caco-2 cells were cultured in trans-wells to confluence and the medium was replaced with culture media from SW620-shGal3 and SW620-shCon (**A**) and HT29-I and -N (**B**) cells and TEER were measured. Caco-2 cells in trans-wells were cultured to confluence and the culture media was either replaced with fresh culture medium (SM) or culture medium from SW620-shGal3 (SW620-shGal3-CM) and SW620-shCon (SW620-shCon-CM) (**C**) or HT29-I (HT29-I-CM) and HT29-N (HT29-N-CM) (**D**) cells with addition of 1 mg/ml FITC-dextran for 0.5 h and the fluorescence intensities in the medium of the bottom wells were measured. **E**, **F** Similar assessments were made as in (**C**, **D**), but a combination of antibodies against MMP-1, MMP-13 and CTSB were added into the conditioned media of SW620-shGal3 and SW620-shCon (**E**) or HT29-I and HT29-N (**F**) cells before they were introduced to Caco-2 monolayers and subsequent analysis of the fluorescence intensity in the bottom wells. Data are presented as mean ± SD of three independent experiments, each in triplicate. ***P < 0.001, **P < 0.01, *P < 0.05 (ANOVA).

It is known that some proteases such as cathepsin-B can degrade cell surface adhesion molecules and disrupt cell–cell contacts [24–26]. For example, cathepsin-B can cleave E-cadherin at the cell surface and disrupt cell–cell junctions of epithelium [27]. To test the influence of galectin-3-induced protease secretion on cell–cell contact and epithelium integrity, Caco-2 cells, which can form tight cell–cell junctions in culture and which are frequently used as a model to study epithelial integrity and the paracellular movement of compounds across an epithelium [28], were cultured to form a tight monolayer in trans-wells. Monolayer integrity was monitored after introduction of conditioned media containing different amounts of proteases induced by galectin-3. It was found that introduction of the conditioned medium from SW620-shCon cells caused a significant reduction in the transepithelial electric resistance (TEER) in comparison to that from SW620-shGal3 cells (Fig. 6A). Introduction of conditioned medium from HT29-I cells also resulted in reduction of cell TEER in comparison to that from HT29-N cells (Fig. 6B). This indicates that galectin-3-mediated protease secretion disrupts tumour cell–cell contacts.

To further assess the impact of galectin-3-mediated protease secretion on epithelial cancer cell monolayer integrity, FITC-dextran was introduced as a paracellular transport marker in the assessment. Penetration of FITC-dextran through the Caco-2 monolayer to the bottom of the trans-wells was seen to be two-fold higher when the cells were cultured in conditioned medium from SW620-shCon cells than that from SW620-shGal3 cells (Fig. 6C). The penetration of FITC-dextran through the Caco-2 monolayer was also seen to be 1.5-fold higher when the cells were cultured in conditioned medium from HT29-I cells than from HT29-N cells (Fig. 6D). In comparison to the cells cultured in normal medium, introduction of 1000 pg/ml exogenous recombinant cathepsin-B or MMP-13 also significantly increased FITC-dextran penetration through the Caco-2 monolayer (Fig. 6C, D). Moreover, introduction of a combination of antibodies against MMP-1, MMP-13 and cathepsin-B significantly reduced FITC-dextran penetration induced by culture in CM from SW620-shcon (Fig. 6E) and HT29-I (Fig. 6F) cells, but had minimal effect on that cultured in CM from SW620-shGal3 and HT29-N cells, respectively. Together, these results indicate that galectin-3-induced protease secretion causes significant disruption of cell–cell contact and increases epithelial cell monolayer permeability and leakage.

### Galectin-3 induces protease secretion through activation of PYK2-GSK3α/β signalling

**Fig. 7:**
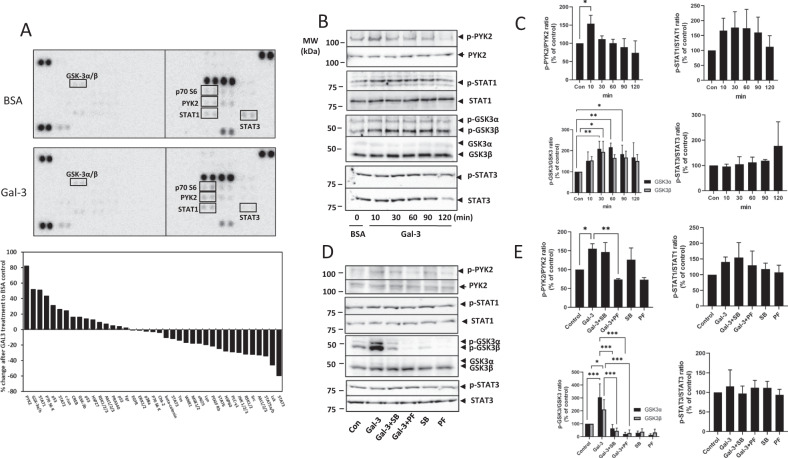
Galectin-3 expression induces activation of PYK2, STAT1 and GSK3α/β signalling. Expression of 37 protein kinases in SW620 cells in response to 10 µg/ml galectin-3 or BSA for 0.5 h was assessed by Proteome Profiler Human Phospho-Kinase Array (**A**, Percentage changes of the kinases in cell response to galectin-3 in comparison to control are shown at the bottom panel). The presence of galectin-3 increases the phosphorylation of PYK2, GSK3α/β, and STAT1 and decreases phosphorylation of STAT3. SW620 cells treated with 10 µg/ml galectin-3 for different times were assessed by immunoblotting using antibodies against p-PYK2, p-STAT-1, p-GSK3α/β or p-STAT-3 (**B**). The blots were striped and reprobed with antibodies against PYK2, STAT-1, GSK3α/β or STAT-3. The band density was quantified and expressed as percentages of phospho-/non-phosphorylated proteins (**C**). In **D** and **E**, SW620 cells were treated with 10 µg/ml galectin-3 or BSA followed by introduction of GSK3α/β inhibitor SB 216763 (SB) or PKY2 inhibitor PF-431396 (PF) for 15 min and the levels of phosphorylated PYK2, STAT-1, GSK3α/β or STAT-3 were analysed by immunoblotting. The blots were striped and reprobed with antibodies against PYK2, STAT-1, GSK3α/β or STAT-3. The densities of the blots from three independent experiments were quantified and are expressed as the percentage of phosphorylated/non-phosphorylated levels of each protein. ***P < 0.001, **P < 0.01, *P < 0.05 (ANOVA).

To gain insight into the molecular mechanism of galectin-3-mediated protease secretion, the phosphorylation profile of 37 key signalling proteins in SW620 cell response to galectin-3 was analysed using a Proteome Profiler Human Phospho-Kinase Array. Galectin-3 treatment led to changes in the expression of several kinases (Fig. 7A). Phosphorylation of four kinases, namely Protein tyrosine kinase 2 (PYK2), Glycogen synthase kinase-3 (GSK3α/β), Signal transducer and activator of transcription 1 (STAT1) and Signal transducer and activator of transcription 3 (STAT3) showed over 50% changes in response to galectin-3. Three of these kinases, PYK2 (82%), GSK3α/β (52%) and STAT1(51%), showed increases in phosphorylation while STAT3 showed a decrease (59%) in phosphorylation.

To confirm the effects of galectin-3 on activation of these kinases indicated by the protein array, SW620 cells were treated with galectin-3 for various times and the phosphorylation status of each of the kinases was measured. Galectin-3 treatment caused time-dependent and rapid increases in the activation of PYK2, STAT1 and GSK3α/β, while phosphorylation of STAT3 was unaffected (Fig. 7B, C). Introduction of the GSK3α/β inhibitor SB216763 (10 µM) decreased the galectin-3-associated increase in GSK3α/β phosphorylation but had little effect on galectin-3-associated phosphorylation of PYK2, STAT1 and STAT3 (Fig. 7D, E). The presence of the PYK2 inhibitor PF431396 (10 µM) on the other hand reduced phosphorylation of both PYK2 and GSK3α/β but caused no/little effect on STAT1 and STAT3 phosphorylation (Fig. 7D, E). These results suggest that activation of GSK3α/β and PYK2 are both involved in the cell response to galectin-3, and PYK2 activation is probably an upstream mediator of GSK3α/β in galectin-3-mediated signalling transduction.

**Fig. 8:**
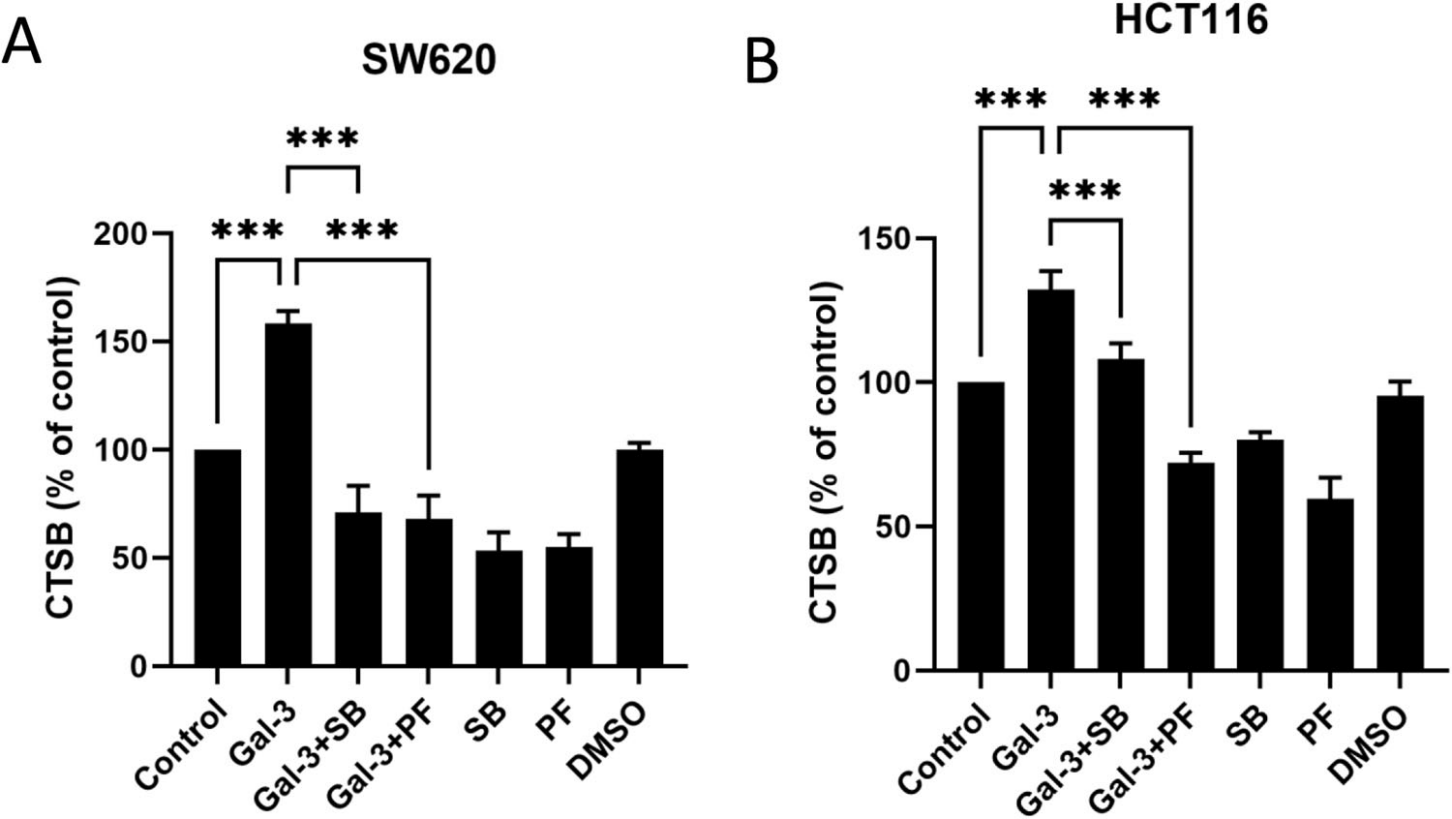
Galectin-3 induces CTSB secretion through PYK2-GSK3α/β activation. SW620 (**A**) and HCT116 (**B**) cells were treated with 10 µg/ml galectin-3 or BSA without or with SB216763 (SB), PF431396 (PF) or DMSO overnight. The concentrations of cathepsin-B in the supernatants were analysed by ELISA. Data are presented as mean ± SEM of three independent experiments. ***P < 0.001, **P < 0.01, *P < 0.05 (ANOVA).

To investigate the relationship between PYK2 and GSK3α/β activation and protease secretion mediated by galectin-3, cathepsin-B secretion in SW620 and HCT116 cells were analysed in the presence or absence of galectin-3, PYK2 inhibitor SB216763 and GSK3α/β inhibitor PF431396. The galectin-3-induced secretion of cathepsin-B was shown to be completely inhibited by the presence of 10 µM PF431396 in both SW620 (Fig. 8A) and HCT116 (Fig. 8B) cells. The presence of SB216763 at this concentration also completely abolished galectin-3-induced cathepsin-B secretion in SW620 cells but only partly inhibited that in HCT116 cells at this concentration. These results suggest that PYK2-GSK3α/β activation is critically involved in galectin-3-mediated secretion of proteases, at least for cathepsin-B in those cells.

## Discussion

This study shows that galectin-3 expression and secretion by human colon cancer cells induces cell secretion of several proteases in an autocrine or paracrine manner through activation of PYK2-GSK3α/β signalling. The secretion of these proteases enhances cancer cell invasion through basement proteins, as well as reduces cancer cell–cell contact and increases epithelium monolayer permeability.

Over 900 protease genes are estimated to exist in the human genome [29]. Under normal physiological conditions, the amount of these proteases is low and tightly controlled [30] and their proteolytic actions contribute to the maintenance of homeostasis [1]. Overexpression of proteases occurs in various cancers [2], and facilitates tumour cell invasion and spreading [31]. It is believed that degradation of ECM by various proteases [32] is a very early step in tumour cell invasion at primary tumour sties [33]. In this study, the secretion of several proteases including cathepsin-B, MMP-1 and MMP-13 by cancer cells was shown to be significantly increased in response to the presence of galectin-3. Cathepsin-B is overexpressed in various cancers such as breast, colorectal, gastric, lung, prostate cancer and gliomas [34]. It is particularly highly expressed at the invasive edge of the tumours [35, 36] and promotes cancer cell invasion [37]. MMP-1 and MMP-13 are primarily responsible for ECM degradation in cancer progression [38]. MMP-1 and MMP-13 are both often overexpressed in epithelial cancers such as breast, prostate, and gastric cancer [32, 39]. They degrade extracellular matrix proteins and promote tumour cell invasion, angiogenesis, and metastasis [12]. The discovery in this study that galectin-3 increases the secretion of Cathepsin-B, MMP-1, and MMP-13 by cancer cells suggests that increasing the secretion of proteases is probably one of the mechanisms behind galectin-3-mediated cancer promotion reported in many previous studies [20, 40, 41].

Galectin-3 is now well recognized as a multi-functional promoter of cancer progression and metastasis [42]. Galectin-3 is found intracellularly as well as extracellularly [21]. Cytoplasmic galectin-3 interacts with Bcl-2 and prevents cell apoptosis [43]. Binding of extracellular galectin-3 to cell surface glycans on growth factors [44], death receptors [45] and adhesion molecules [46] promotes cancer cell adhesion, invasion, angiogenesis, and tumour cell escape from immune surveillance [47].

Galectin-3 has previously been reported to increase secretion of MMP-1 and −9 in melanoma cells [48] by binding to lysosome-associated membrane protein-1 (LAMP1) [49, 50]. It has also been reported to increase MMP-1 expression and enhance invasion of gastric cancer cells [51]. The present study shows that the presence of galectin-3 induces secretion of a number of proteases including cathepsin-B, MMP-1 and MMP13. This implies that the protease secretion mediated by galectin-3 in different types of cancers may not be always the same and may depend on the expressions of particular galectin-3 binding glycans/receptors in different types of cancers. Many cell surface glycoproteins such as growth factor receptors, adhesion and signalling proteins are known to be recognized by galectin-3 [52] and are differentially expressed by different cancer types.

This study has shown that galectin-3-induced protease secretion decreases epithelial monolayer integrity. Epithelium monolayer integrity is crucial in maintaining tissue homeostasis [53]. Disruption of epithelial integrity is a critical step in primary tumour cell invasion [54]. E-cadherin is a key cell adhesion molecule for maintaining tight cell–cell junctions in the epithelium and can be proteolytically cleaved by cathepsins [27]. Cathepsin-B can also bind to the annexin II heterotetramer (AIIt) and activates other proteases such as matrix metalloproteinases and urokinase plasminogen activator (uPA) and indirectly disrupts epithelial cell–cell contact and monolayer integrity [55–57]. The increased cancer cell secretion of cathepsin-B by galectin-3 reported in this study can therefore decrease epithelium integrity and aid tumour cell break up at primary tumour sites.

The present study has also demonstrated that galectin-3-induced protease secretion enhances tumour cell invasion through basement proteins. The basement proteins underneath the epithelium are rich in protease substrates and can be catalytically digested by proteases such as cathepsin-B [58], MMP-1 [59] and MMP-13 [32]. Cathepsin-B can cleave laminin, fibronectin, type IV collagen and tenanscin-C in matrix proteins to aid tumour cell invasion [60]. MMP-1 and MMP-13 can also directly degrade type I and III collagens enriched in ECM [12]. The increased secretion of those proteases by galectin-3 in the tumour microenvironment therefore supports tumour cell invasion through the basement in tumour cell spreading.

Activation of several signalling molecules particularly PYK2 and GSK3α/β has been shown in this study to be involved in galectin-3-induced protease secretion. PYK2 activation has been reported previously to regulate cathepsin-B secretion from human primary macrophages during the immune response [61] and their activation have both shown to enhance cancer cell invasion and metastasis [62, 63]. A rapid increase and decrease of PYK2 activation in breast cancer cells has also been shown to increase PYK2 complex formation with p190 RhoGAP (p190), RasGAP, ErbB-2, and Src, leading to activation of the MAPK signalling and increase of cancer cell invasion [64]. GSK3 activation has previously been reported to increase proliferation and survival of ovarian cancer cells [65]. Administration of GSK3β inhibitors showed to suppress cell proliferation and inhibit tumour formation in mice [65, 66]. Thus, activation of PYK2 and GSK3 signalling in cancer cells by galectin-3 may itself influence cancer progression in addition to its effect through induction of protease secretion.

Overall, this study suggests an important mechanism in galectin-3-mediated promotion of cancer progression. It provides further evidence to the increased realization of galectin-3 as a potential therapeutic target for cancer treatment.

## Supplementary information

Supplementary figures

Original Data File

Reproducibility checklist

## Data Availability

All datasets generated and analysed during this study are included in this published article and its Supplementary Information files. Additional data are available from the corresponding author on reasonable request.
