# Supplementary material for: Galectin-3 promotes secretion of proteases that decrease epithelium integrity in human colon cancer cells
Source: Cell Death Dis. 2023 Apr 13;14(4):268. doi: 10.1038/s41419-023-05789-x (PMC10102123; doi:10.1038/s41419-023-05789-x)
Supplement: Supplementary file 1 — Supplementary figures [file 41419_2023_5789_MOESM1_ESM.pdf]

Fig S1

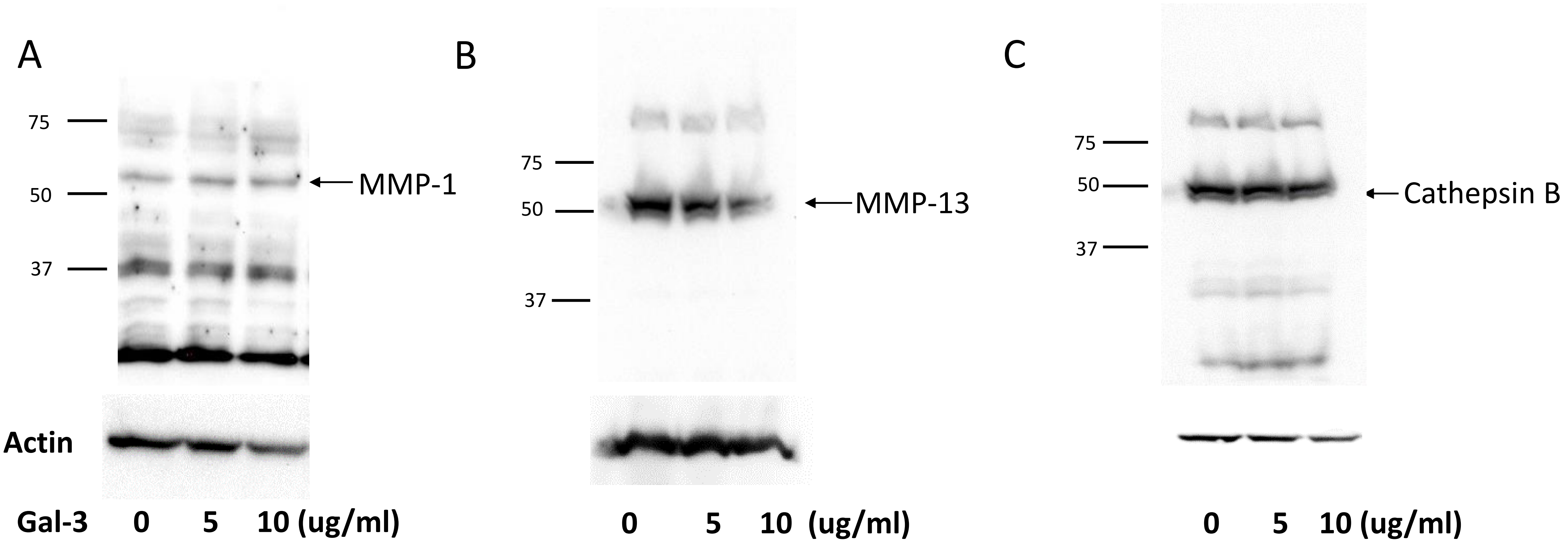

Figure 1S. The presence of galectin-3 does not affect the expressions of Cathepsin-B, MMP-1 and MMP-1 in SW620 cells. SW620 cells were treated with 0, 5, and 10 μg/ml galectin-3 for 24 hr and cell lysates were analysed by immunoblotting.
