# Supplementary material for: Galectin-3 promotes secretion of proteases that decrease epithelium integrity in human colon cancer cells
Source: Cell Death Dis. 2023 Apr 13;14(4):268. doi: 10.1038/s41419-023-05789-x (PMC10102123; doi:10.1038/s41419-023-05789-x)
Supplement: Supplementary file 2 — Original Data File [file 41419_2023_5789_MOESM2_ESM.pptx]

## Slide 1
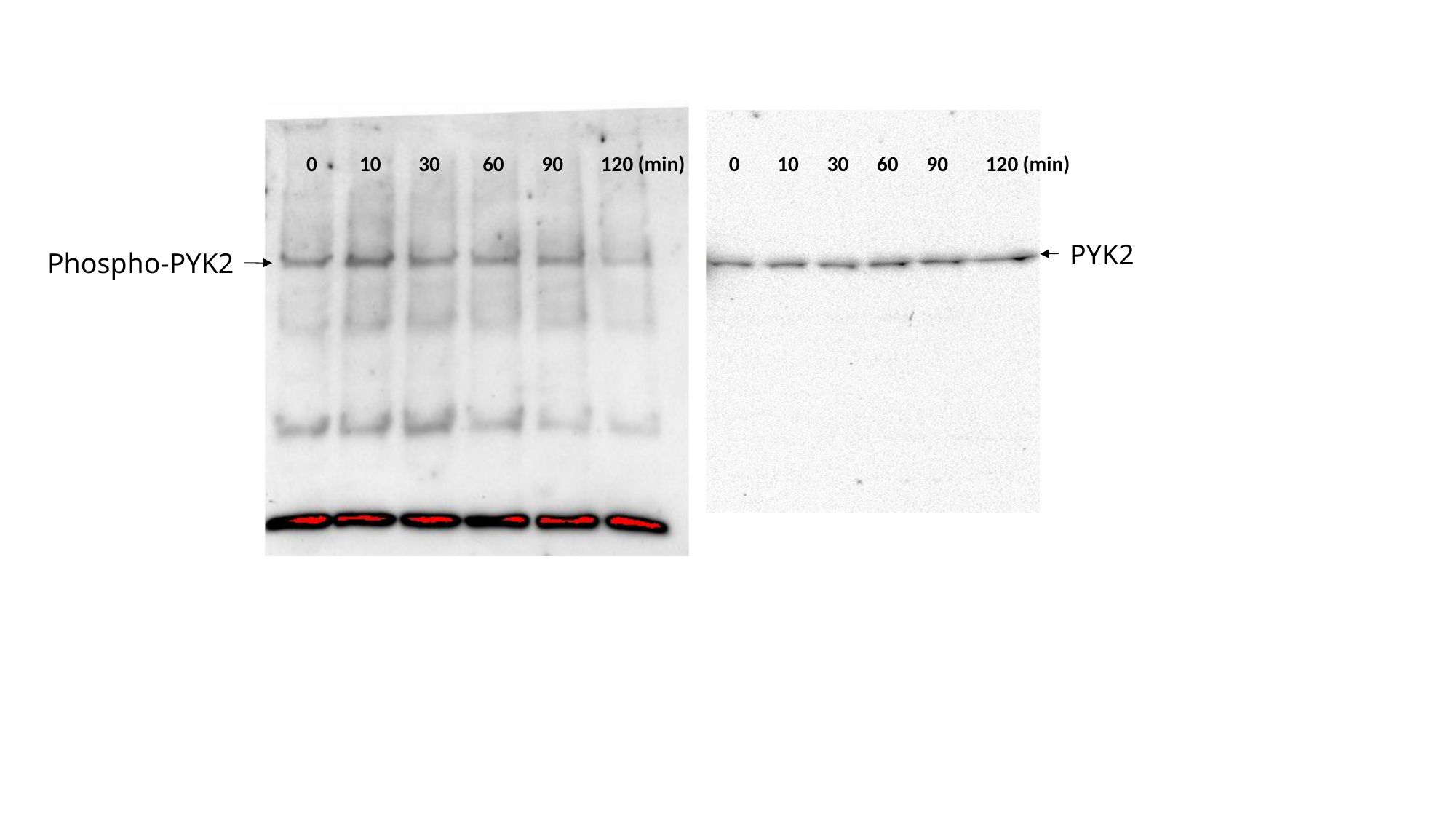

0 10 30 60 90 120 (min)
 0 10 30 60 90 120 (min)
PYK2
Phospho-PYK2

## Slide 2
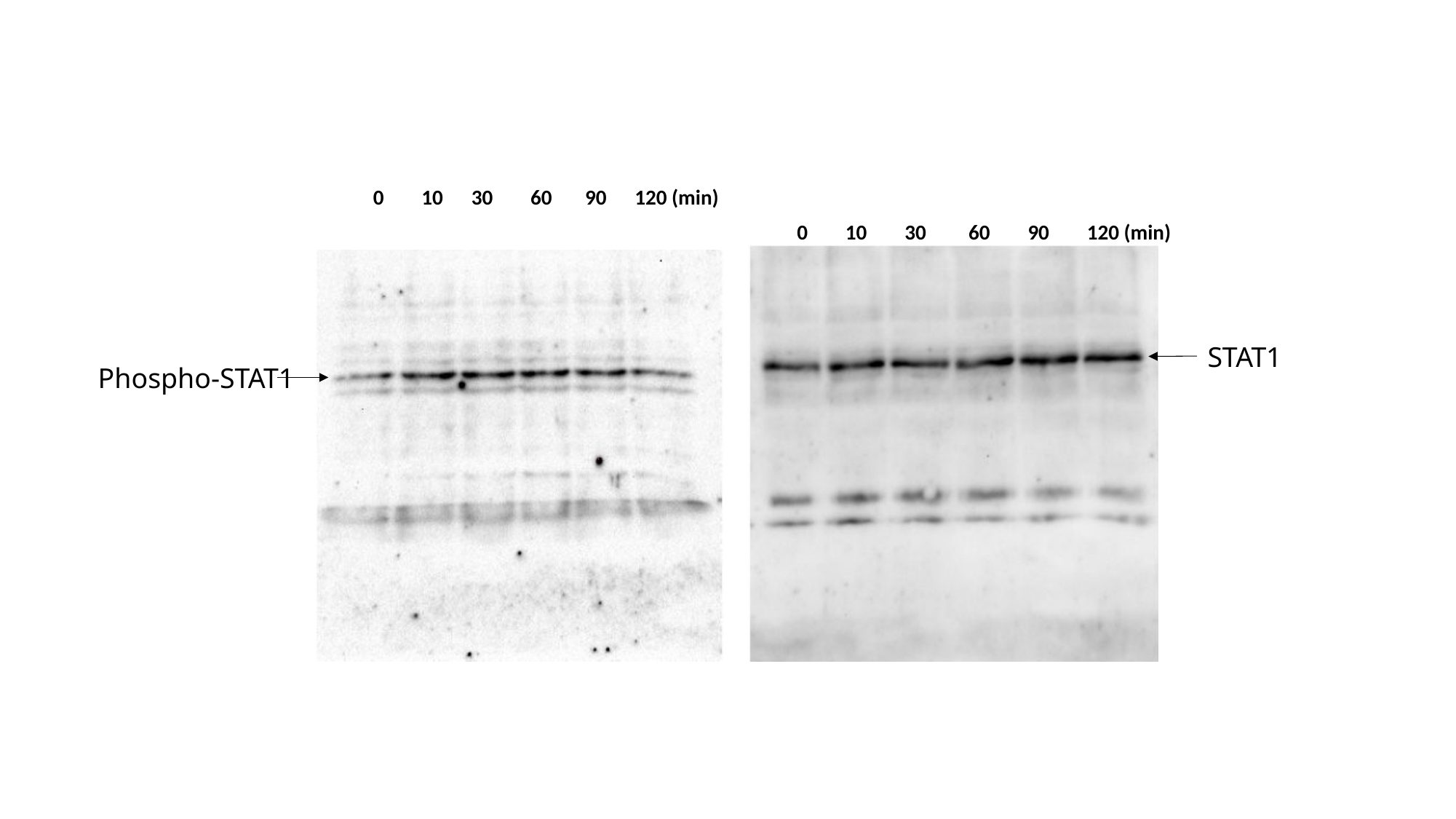

0 10 30 60 90 120 (min)
 0 10 30 60 90 120 (min)
STAT1
Phospho-STAT1

## Slide 3
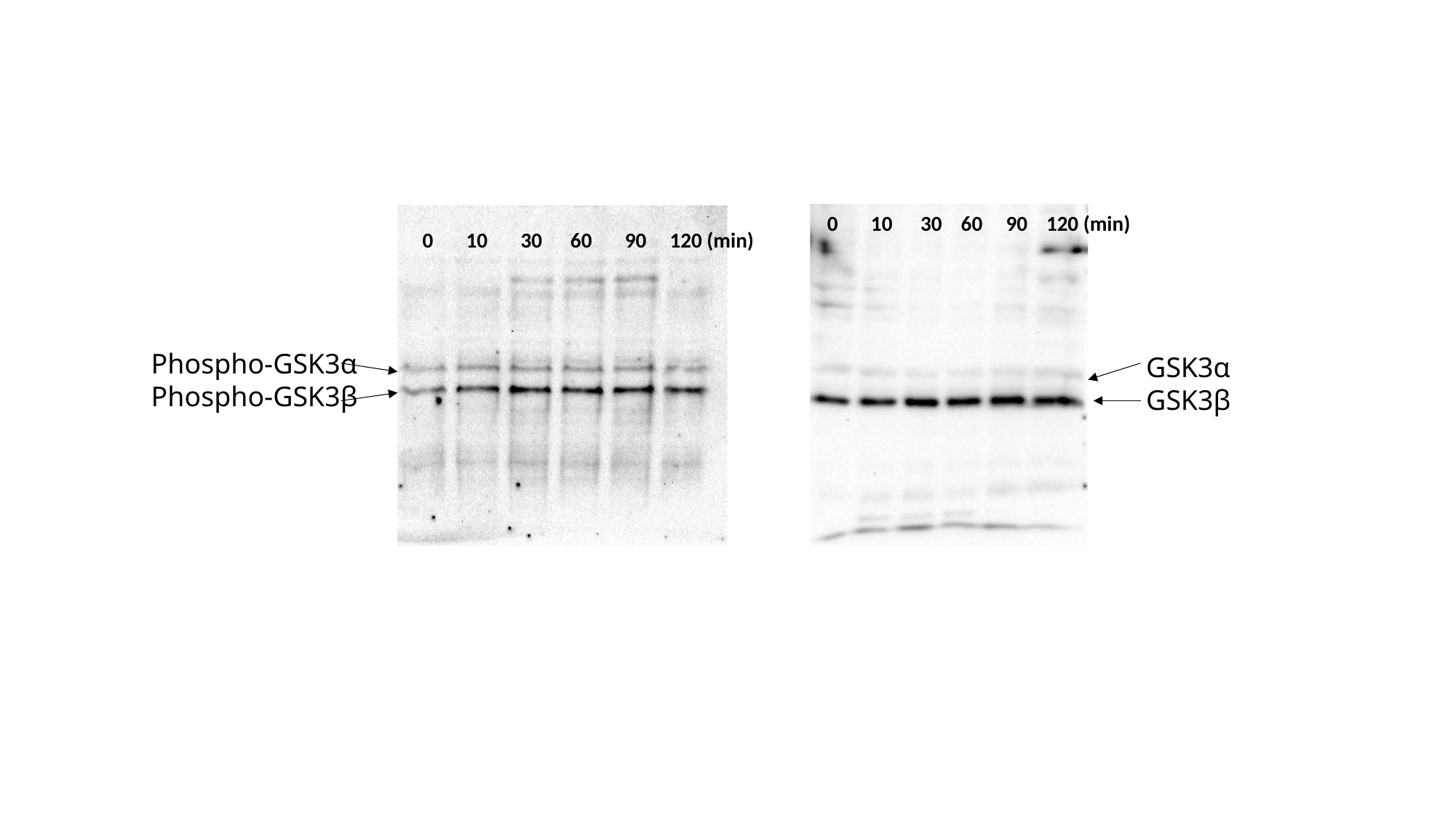

0 10 30 60 90 120 (min)
 0 10 30 60 90 120 (min)
Phospho-GSK3α
Phospho-GSK3β
GSK3α
GSK3β

## Slide 4
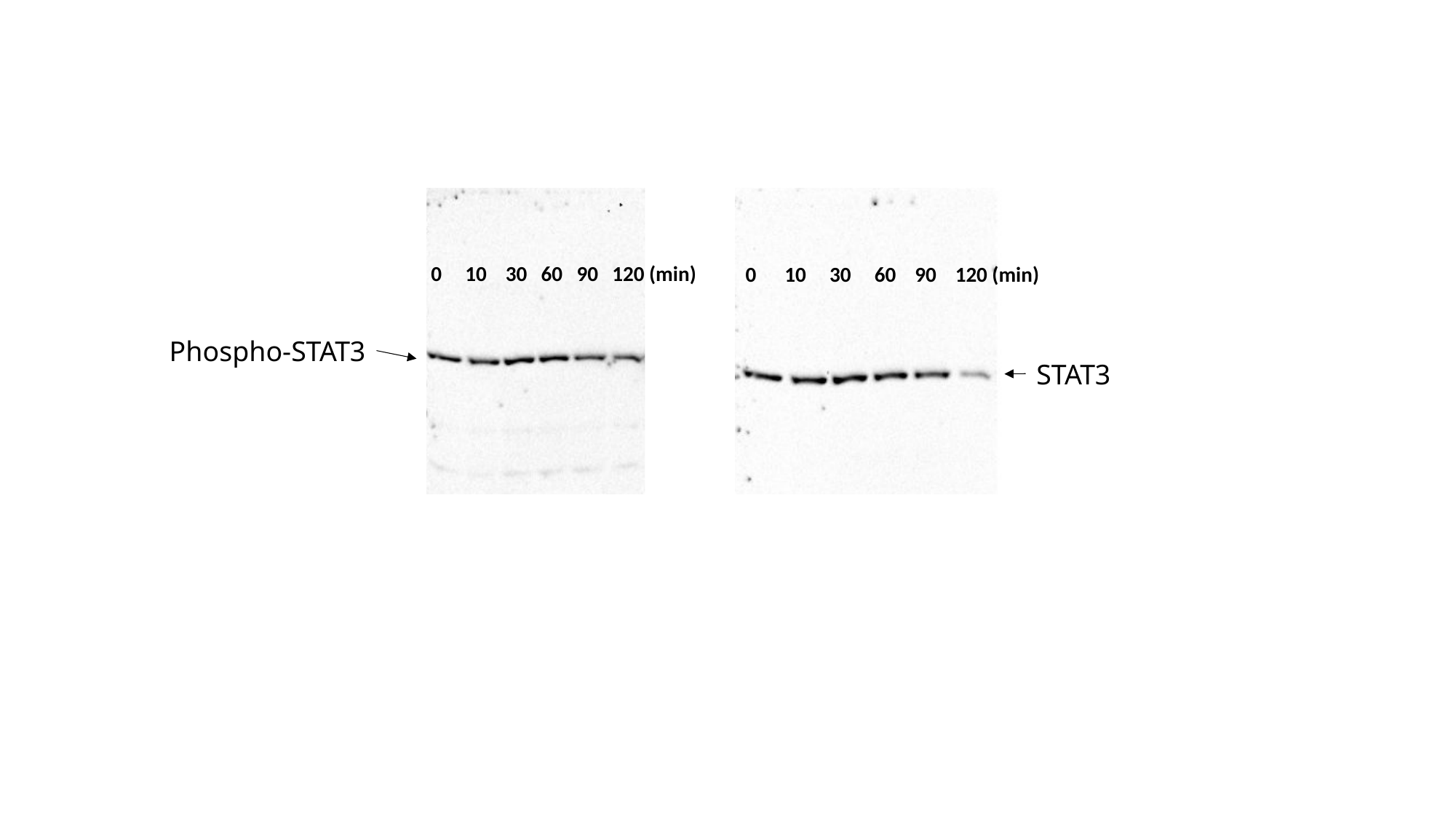

0 10 30 60 90 120 (min)
 0 10 30 60 90 120 (min)
Phospho-STAT3
STAT3

## Slide 5
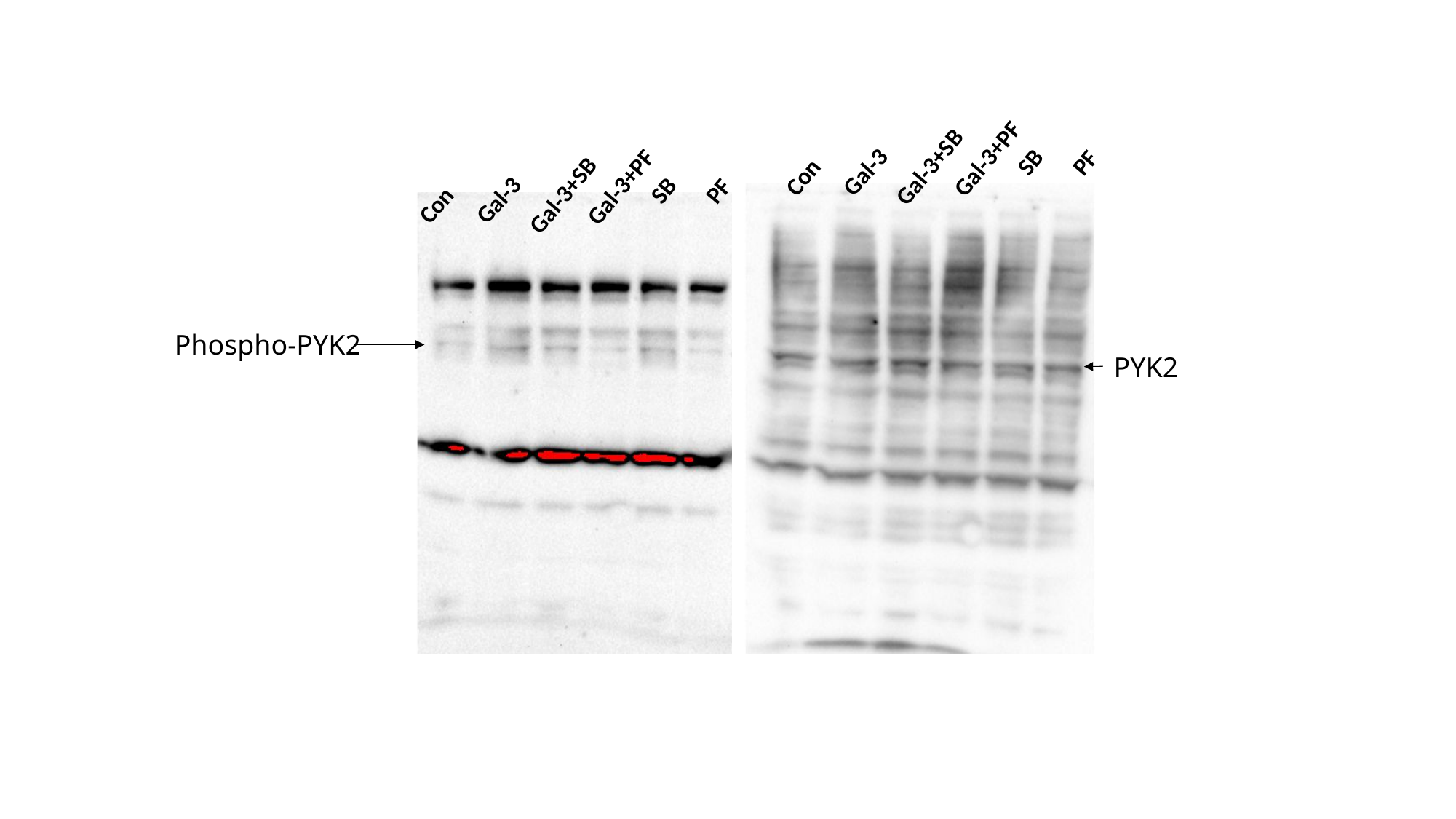

Gal-3
SB
PF
Con
Gal-3+SB
Gal-3+PF
Gal-3
SB
PF
Con
Gal-3+SB
Gal-3+PF
Phospho-PYK2
PYK2

## Slide 6
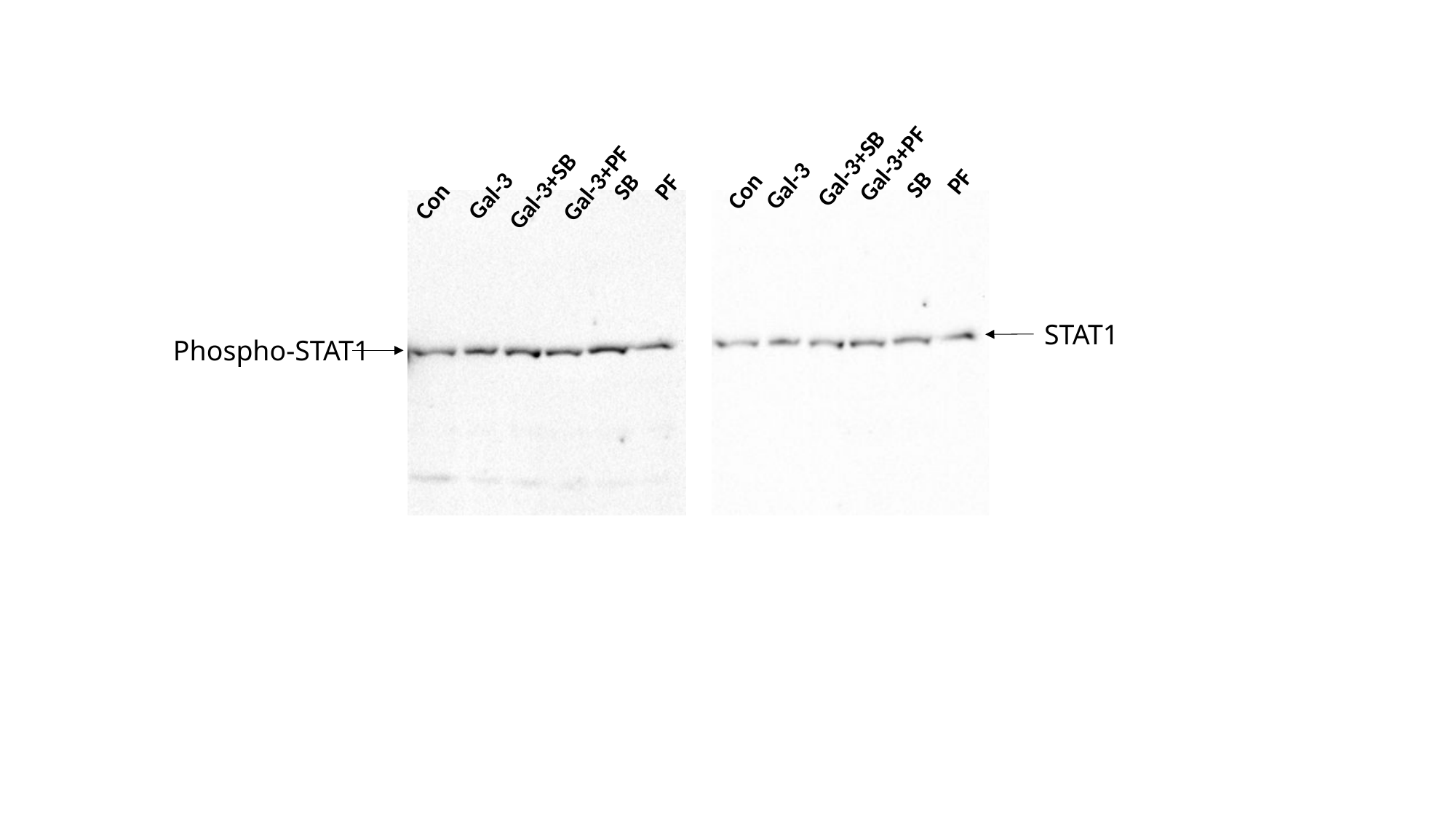

Gal-3
PF
Gal-3+SB
SB
Con
Gal-3
SB
PF
Gal-3+PF
Con
Gal-3+SB
Gal-3+PF
STAT1
Phospho-STAT1

## Slide 7
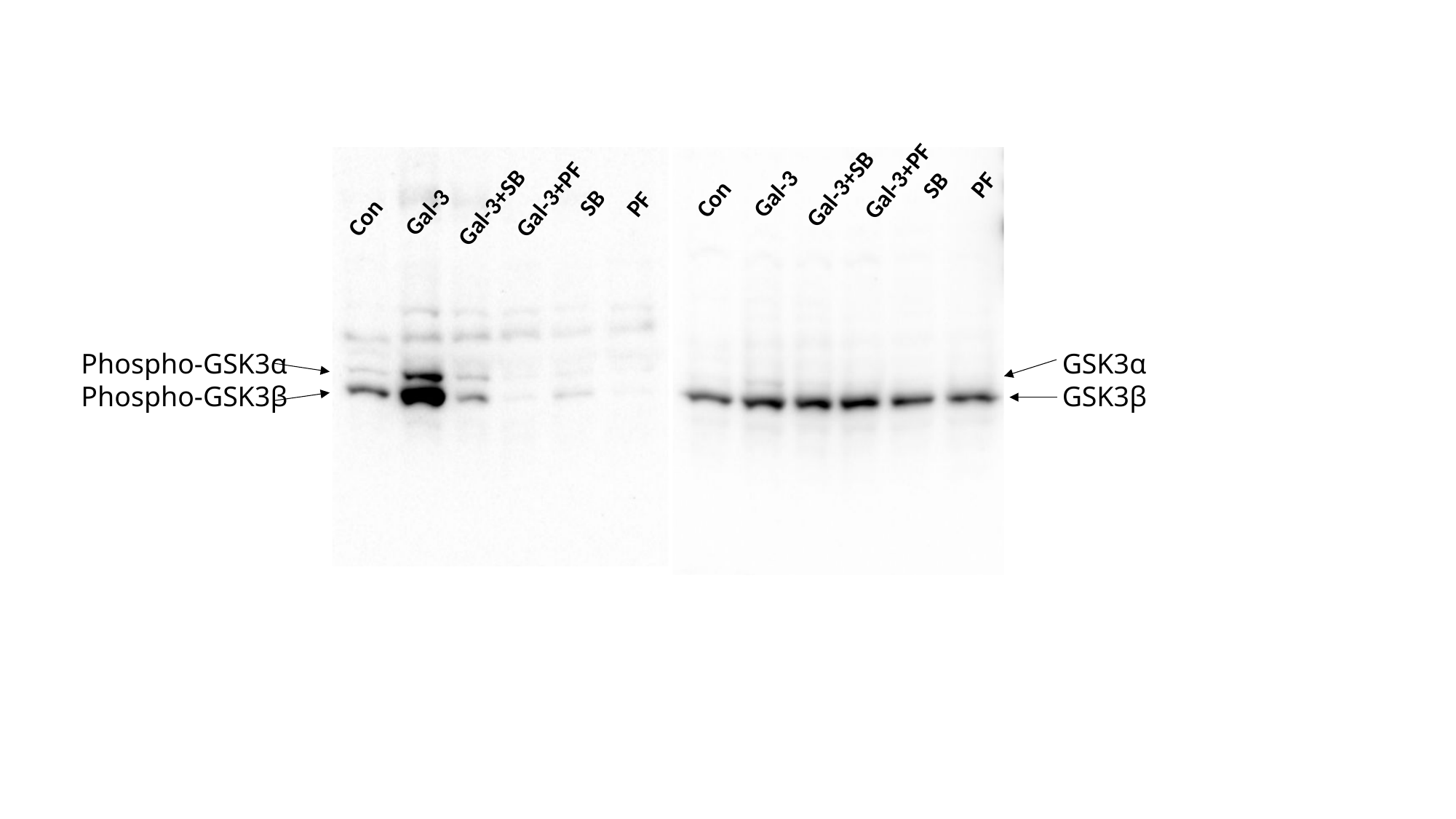

Gal-3
PF
SB
Con
Gal-3
SB
Gal-3+SB
PF
Gal-3+PF
Con
Gal-3+SB
Gal-3+PF
Phospho-GSK3α
Phospho-GSK3β
GSK3α
GSK3β

## Slide 8
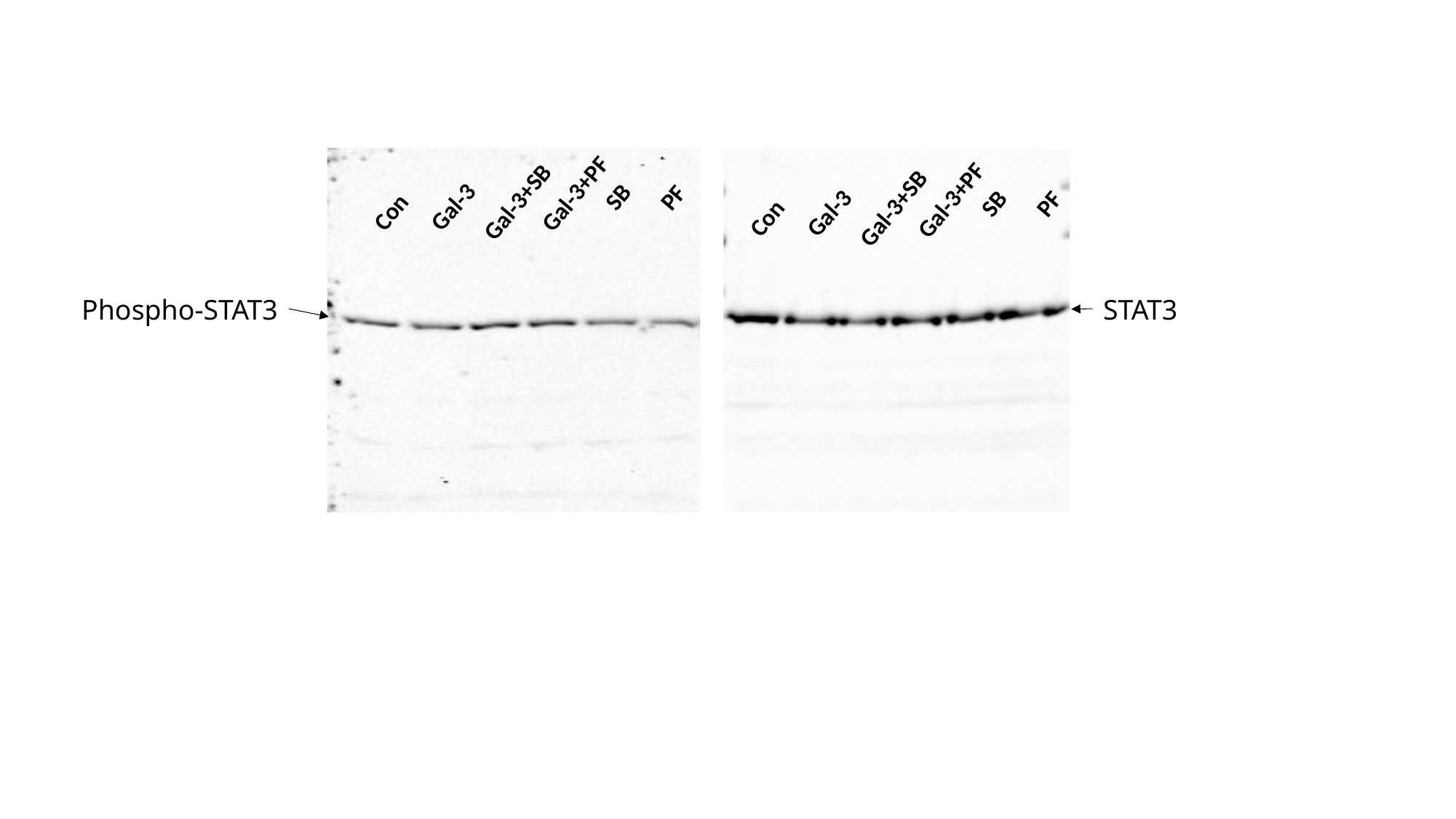

Gal-3
SB
PF
Gal-3
SB
PF
Con
Con
Gal-3+SB
Gal-3+PF
Gal-3+SB
Gal-3+PF
Phospho-STAT3
STAT3
